# The impact of estrogen on benign breast tumors and exploration of recurrence mechanisms

**DOI:** 10.3389/fonc.2025.1609832

**Published:** 2025-07-09

**Authors:** Zhe Fan, Huijin Wang, Yu Liu, Zongshuo Yu, Jingjing Li, Yuan Teng

**Affiliations:** ^1^ Department of Breast and Thyroid Surgery, Guangzhou Women and Children’s Medical Center, Guangzhou Medical University, Guangzhou, Guangdong, China; ^2^ Thyroid and Breast Surgery Department, The Third Affiliated Hospital of Sun Yat Sen University, Guangzhou, Guangdong, China; ^3^ School of Pediatrics, Guangzhou Medical University, Guangzhou, Guangdong, China

**Keywords:** estrogen, benign breast tumors, recurrence mechanisms, hormone dependence, microenvironment

## Abstract

Benign breast tumors are among the most common breast diseases affecting women, with their pathogenesis closely linked to hormonal influences, particularly estrogen. This review offers a comprehensive overview of the mechanisms through which estrogen affects benign breast tumors and examines the role of estrogen in tumor cell growth, proliferation, and apoptosis. Furthermore, this review explores the recurrence mechanisms of benign breast tumors, analyzing factors such as hormone dependence, microenvironmental influences, and genetic susceptibility. By generating relevant literature, this article aims to offer new insights into clinical treatment and prevention strategies aimed at managing benign breast tumors.

## 1 Introduction

Benign breast tumors are among the most common types of breast diseases affecting women, and their incidence is steadily increasing. Estrogen, a critical bioactive substance, has been extensively studied for its role in the occurrence and progression of breast tumors. Although benign tumors are generally considered less aggressive than malignant tumors, tumor recurrence remains a significant concern in clinical practice. Understanding the impact of estrogen on benign breast tumors and the mechanisms underlying their recurrence is crucial for improving patient outcomes and prognoses.

Estrogen has multifaceted influence on breast tissues, affecting cellular proliferation, differentiation, and apoptosis. The presence of estrogen receptors (ER) in breast tissue facilitates these processes, making it a central focus in the study of breast tumors. In benign conditions like fibroadenomas and phyllodes tumors, estrogen may promote cellular proliferation and contribute to tumor growth. Estrogen receptor-positive tumors show a higher propensity for recurrence, suggesting that estrogen signaling pathways play a pivotal role in tumor behavior and recurrence patterns (1, 2). Furthermore, the interplay between estrogen and other hormonal factors, including progesterone and growth factors, can further complicate the pathophysiology of benign breast tumors.

The recurrence of benign breast tumors can be attributed to several factors, including incomplete surgical excision, hormonal influences, and the biological characteristics of the tumor itself. For example, breast fibromatosis, a benign tumor, is known for its local aggressiveness and high recurrence rate despite being non-metastatic (2). Moreover, the role of estrogen in stimulating the growth of benign tumors raises concerns about the implications of hormone replacement therapy and other estrogenic treatments. Estrogen replacement therapy can lead to the growth of benign tumors, such as nipple adenomas, which exemplifies the potential risks associated with hormonal interventions in women undergoing treatment for menopausal symptoms (3).

Taken together, the relationship between estrogen and benign breast tumors is complex and multifactorial. Although benign tumors are often considered less concerning than malignant ones, their high potential for recurrence necessitates a thorough understanding of the underlying mechanisms influenced by estrogen. This knowledge is essential to develop effective management strategies and improve the prognosis of patients with benign breast tumors. Future research should focus on elucidating the molecular pathways involved in estrogen signaling within benign breast tumors and on identifying potential biomarkers that could predict recurrence and thus guide therapeutic interventions.

## 2 Main body

### 2.1 The biological effects of estrogen

Estrogen is a crucial hormone that plays a significant role in various physiological processes, particularly in the reproductive system. It is responsible for the development and regulation of the female reproductive system and secondary sexual characteristics. The influence of estrogen extends beyond the reproductive system, affecting the cardiovascular, skeletal, and central nervous systems. The hormone exerts its effects through specific receptors, primarily estrogen receptors alpha (ERα) and beta (ERβ), which mediate both genomic and non-genomic actions. The complexity of estrogen signaling is underscored by its ability to interact with various signaling pathways, thus impacting cellular functions like proliferation, differentiation, and apoptosis. In addition, the role of estrogen in maintaining bone density, regulating lipid metabolism, and influencing mood and cognitive functions highlights its importance in overall health, particularly in women (4, 5).

#### 2.1.1 Synthesis and metabolism of estrogen

Estrogen synthesis primarily occurs in the ovaries, where androgens are converted into estrogens by aromatase. This process is regulated by a complex interplay of hormones, including luteinizing hormone (LH) and follicle-stimulating hormone (FSH). Besides the ovaries, other tissues and organs, such as adipose tissue, the liver, and the brain, can also produce estrogens, particularly after menopause when ovarian production declines. Estrogen metabolism involves its conversion into various metabolites, which can have distinct biological activities. The primary metabolic pathway is through hydroxylation, leading to the formation of catechol estrogens, which can show both estrogenic and anti-estrogenic properties. The balance between these metabolites is crucial as it can influence the risk of developing estrogen imbalance-related diseases, including breast cancer. Furthermore, the clearance of estrogen from the body is facilitated by the liver, where it undergoes conjugation and is excreted via bile or urine (1, 3).

#### 2.1.2 Types and functions of estrogen receptors

Estrogen exerts its biological effects through two main types of estrogen receptors: ERα and ERβ, each with distinct tissue distribution and functions. ERα is predominantly found in reproductive tissues, such as the uterus and ovaries, and is primarily responsible for mediating the proliferative effects of estrogen. Conversely, ERβ is more widely distributed in non-reproductive tissues, including the brain and cardiovascular system, and is thought to have protective roles against certain diseases. In addition, a third type of receptor, the G protein-coupled estrogen receptor (GPER), has been identified, which mediates rapid non-genomic effects of estrogen. The activation of these receptors triggers various signaling cascades that can lead to changes in gene expression, cellular growth, and differentiation. The differential expression and activation of these receptors in various tissues underscore the complexity of estrogen signaling and its implications in health and disease, particularly in the context of hormone-dependent cancers (4, 6, 7).

### 2.2 The relationship between estrogen and benign breast tumors

The relationship between estrogen and benign breast tumors has garnered significant attention in recent years, particularly due to the hormone’s role in cell proliferation and the tumor microenvironment. Estrogen, primarily through its receptors, influences various cellular processes, including growth and differentiation, which are critical to the development of both benign and malignant tumors. Estrogen can reportedly stimulate the proliferation of breast epithelial cells, which may contribute to the formation of benign tumors like fibroadenomas and adenomas (8). For instance, a study highlighted that in a patient with a nipple adenoma, the proliferation of ductal structures was attributed to estrogen replacement therapy, suggesting a direct correlation between estrogen exposure and the growth of benign tumors (3). Furthermore, the presence of estrogen receptors in benign tumors implies that these tumors may respond to hormonal fluctuations, potentially leading to increased growth rates during periods of elevated estrogen levels, such as during pregnancy or hormone replacement therapy.

#### 2.2.1 The impact of estrogen on tumor cell proliferation

The impact of estrogen on tumor cell proliferation is a critical factor in understanding the development of benign breast tumors. The hormone primarily exerts its effects through estrogen receptors (ERα and ERβ), which, upon binding with estrogen, activate various signaling pathways that promote cell division and growth. Studies have shown that estrogen can enhance the proliferation of breast epithelial cells, which is a fundamental characteristic of tumorigenesis (9–11). For instance, the expression of estrogen receptors has been observed in benign breast lesions, indicating that these tissues are responsive to estrogen (1). Moreover, the estrogen-mediated activation of pathways like the PI3K/Akt pathway and MAPK pathway further underscores its role in promoting cellular proliferation. This is particularly relevant in the context of benign tumors as excessive stimulation of these pathways can lead to hyperplasia and tumor formation. In addition, the use of aromatase inhibitors in clinical settings has shown that reducing estrogen levels can effectively decrease the proliferation of estrogen-dependent tumors, upholding the notion that estrogen plays a pivotal role in tumor cell growth (12). Thus, understanding the mechanisms by which estrogen influences cell proliferation is essential to developing strategies to manage benign breast tumors.

#### 2.2.2 Regulation of the breast tissue microenvironment by estrogen

Estrogen not only influences tumor cell proliferation but also plays a crucial role in modulating the breast tissue microenvironment, which is integral to tumor development and progression. The breast microenvironment comprises various cell types, including stromal cells, adipocytes, and immune cells, all of which interact with tumor cells and can influence their behavior. Estrogen reportedly alters the composition and functionality of these stromal cells, promoting a pro-tumorigenic environment. For example, estrogen can enhance the secretion of growth factors and cytokines from stromal cells, thus creating a paracrine signaling network that supports tumor cell proliferation and survival (13). In addition, the hormone can influence the metabolic activity of adipocytes within the breast tissue, leading to increased local estrogen production through the conversion of androgens to estrogens, thus further fueling tumor growth (14). This interplay between estrogen and the breast microenvironment highlights the complexity of benign tumor development as changes in the microenvironment can contribute to tumor initiation and progression. Understanding these interactions is crucial for developing targeted therapies that can disrupt the supportive roles of the microenvironment in benign breast tumors, thus potentially reducing their incidence and progression. The extracellular matrix (ECM) provides structural support to tissues and plays a vital role in cell signaling. This remodeling can alter the physical properties of the tumor microenvironment, affecting cell adhesion, migration, and communication between tumor cells and surrounding stromal cells. Changes in ECM composition and organization can enhance the invasive potential of benign tumors and may contribute to their recurrence by creating a more favorable environment for tumor regrowth.

### 2.3 Mechanisms of recurrence in benign breast tumors

The recurrence of benign breast tumors, such as fibroadenomas, is a complex phenomenon influenced by various biological mechanisms. Understanding these mechanisms is crucial to develop effective management strategies and predict patient outcomes. Recurrence can occur because of several factors, including hormonal influences, the tumor microenvironment, and genetic predispositions. Hormonal factors, particularly estrogen and progesterone, play a significant role in the proliferation of breast tissue and can contribute to the recurrence of benign tumors. The interaction of these hormones with specific receptors on tumor cells can stimulate growth and lead to tumor recurrence after initial treatment. Furthermore, the microenvironment surrounding the tumor, including stromal cells and extracellular matrix components, can also influence tumor behavior and recurrence rates. Recent studies have highlighted the importance of understanding the interplay among these factors to better predict and manage the recurrence of benign breast tumors (15–17).

#### 2.3.1 Hormone-dependent recurrence

Hormonal factors are pivotal in the recurrence of benign breast tumors, particularly estrogen receptor-positive tumors. The growth of benign tumors, such as fibroadenomas, is typically stimulated by estrogen, which promotes cellular proliferation and differentiation. In patients with a history of benign breast disease, fluctuations in hormone levels, particularly during menstrual cycles, pregnancy, and menopause, can change tumor growth dynamics. Elevated levels of circulating estrogen can reportedly stimulate the proliferation of benign breast tissue, potentially leading to recurrence. For example, the presence of estrogen receptors in benign lesions indicates a hormonal influence that can facilitate tumor growth and recurrence (16, 18). Besides, the use of hormonal therapies, such as estrogen replacement therapy during menopause, has been associated with an increased recurrence risk in women with a history of benign breast tumors. Therefore, carefully monitoring hormonal levels and considering the implications of hormonal therapies are essential for managing patients with benign breast tumors to minimize the recurrence risk (19, 20).

#### 2.3.2 Influence of microenvironmental factors on recurrence

The microenvironment around benign breast tumors significantly influences their behavior and potential for recurrence. This microenvironment comprises various cellular components, including fibroblasts, immune cells, and extracellular matrix proteins, which interact with tumor cells and can modulate their growth and survival. For example, the presence of inflammatory cells in the tumor microenvironment can promote a pro-tumorigenic environment that fosters tumor recurrence. In addition, factors like hypoxia and nutrient availability can alter the tumor microenvironment, impacting tumor cell metabolism and growth dynamics. Research has shown that the interaction between tumor cells and the surrounding stroma can result in changes in gene expression that favor tumor recurrence (16, 18). Furthermore, the signaling pathways activated by these interactions, such as those involving growth factors and cytokines, can enhance the invasive potential of benign tumors and consequently lead to recurrence. Understanding these microenvironmental influences is crucial to develop targeted therapies aimed at disrupting these interactions and preventing the recurrence of benign breast tumors (17, 21).

### 2.4 Genetic susceptibility and benign breast tumors

Genetic susceptibility significantly influences the development of benign breast tumors, which are widely characterized by their non-invasive nature but can exhibit complex biological behaviors. Several studies have identified specific genetic alterations associated with benign breast tumors like fibroadenomas and nipple adenomas. For instance, mutations in the MED12 gene have been found to be prevalent in fibroadenomas, highlighting as intended genetic basis underlying these common benign lesions (22). Furthermore, the presence of estrogen receptors in tumors like nipple adenomas suggests that hormonal influences also synergistically interact with genetic predispositions to further exacerbate tumor growth (3). The interplay between genetic factors and hormonal environment underscores the complexity of benign breast tumors, indicating that both intrinsic genetic mutations and extrinsic hormonal stimuli contribute to tumor formation.

#### 2.4.1 The role of genetic factors in benign tumors

Genetic factors significantly contribute to the pathogenesis of benign breast tumors, influencing their development and recurrence. Research has shown that specific genetic mutations, such as those in the MED12 gene, occur frequently in benign breast tumors like fibroadenomas, suggesting a common genetic pathway in their formation (22). In addition, the expression of matrix metalloproteinases (MMPs), particularly MMP-13, has been linked to the biological behavior of both benign and malignant breast lesions, indicating that genetic alterations can influence tumor characteristics and potentially their clinical outcomes (1). Furthermore, the identification of recurrent mutations in benign tumors raises questions about their potential to progress to malignancy, thus emphasizing the need for future genetic research to comprehensively study the implications of these mutations in benign breast tumors.

#### 2.4.2 The association between genetic mutations and tumor recurrence

The recurrence of benign breast tumors is a critical concern, and emerging evidence suggests that specific genetic mutations may be associated with it. Benign tumors can reportedly harbor genetic alterations that predispose them to recurrence, suggesting that the genetic landscape of these tumors influences their clinical behavior (23). For instance, fibroadenomas reportedly show a range of somatic mutations, with some studies suggesting that the presence of certain mutations correlates with a higher likelihood of recurrence (22). In addition, the role of hormonal therapy in the context of benign tumors like nipple adenomas raises further questions about how genetic predispositions may interact with treatment modalities to affect recurrence rates (3). Understanding these associations is crucial to develop targeted surveillance strategies and therapeutic approaches for patients with benign breast tumors.

### 2.5 Future research directions and clinical applications

The exploration of future research directions and clinical applications in breast cancer treatment is crucial for improving patient outcomes. Focusing on estrogen modulation and innovative preventive strategies can significantly improve the management of hormone receptor-positive breast cancer, which is widely prevalent among patients. This section delves into the potential applications of estrogen modulators and new preventive strategies to combat cancer recurrence.

#### 2.5.1 Potential applications of estrogen modulators

Estrogen modulators, such as selective estrogen receptor modulators (SERMs) and selective estrogen receptor downregulators (SERDs), have gained attention for their potential in treating estrogen receptor-positive (ER^+^) breast cancer. These agents work by either activating or inhibiting estrogen receptors, thus influencing tumor growth and progression. Current research highlights the need for further investigation into the mechanisms of action of these modulators, particularly in overcoming resistance to endocrine therapies, which remains a significant challenge in breast cancer management (24). The development of novel SERMs and SERDs that can selectively target cancerous tissues while minimizing adverse effects is a promising avenue for future studies. In addition, combining these agents with other therapeutic modalities, such as CDK4/6 inhibitors or PI3K/AKT/mTOR pathway inhibitors, may show a synergistic effect with a more comprehensive treatment approach (25). Furthermore, a better understanding of the role of estrogenic alkaloids and phytoestrogens in modulating estrogen pathways could open new avenues for therapeutic interventions, particularly in patients resistant to traditional hormone therapies (26). Overall, the potential applications of estrogen modulators in clinical settings warrant extensive research to optimize their use in breast cancer treatment. While SERMs have established efficacy in the treatment of malignant breast tumors, their role in benign breast tumors remains contentious.

#### 2.5.2 New strategies for preventing recurrence

Preventing recurrence in breast cancer patients, particularly in those with ER^+^ tumors, is a critical area of research. Current strategies focus on the use of extended endocrine therapy, which has been shown to significantly reduce the risk of recurrence (27). However, the challenge remains in identifying patients who would benefit the most from prolonged therapy while minimizing adverse effects associated with long-term hormone deprivation. To this end, innovative approaches, such as the use of biomarkers to predict treatment response and recurrence risk, are being explored (28). In addition, the integration of lifestyle modifications, such as weight management and physical activity, into treatment plans may further reduce recurrence rates (29). Another promising strategy involves the use of immunotherapy and targeted therapies that can complement traditional endocrine treatments, particularly in patients resistant to standard therapies (30). Developing personalized treatment regimens that consider individual tumor biology and patient characteristics is essential for improving outcomes and reducing the recurrence risk. Future research should focus on validating these strategies in clinical trials to establish their efficacy and safety in diverse patient populations.

## 3 Conclusion

The role of estrogen in the onset and development of benign breast tumors is significant and multifaceted. As we have comprehensively explored in this review, estrogen influences tumorigenesis through various mechanisms, such as hormonal dependency, changes in the microenvironment, and genetic susceptibility. The complexity of these interactions necessitates a nuanced understanding of how estrogenic activity contributes to the onset and recurrence of benign breast tumors.

Clinically, the recurrence of benign breast tumors is a significant concern, and the understanding of recurrence patterns can be closely linked to the molecular mechanisms of estrogen signaling. Estrogen receptor-positive benign tumors exhibit a higher propensity for recurrence, particularly in patients with elevated estrogen levels. This correlation suggests that the presence and activity of estrogen receptors in these tumors may directly influence their growth dynamics and likelihood of regrowth after surgical excision.

Future research should aim to clarify the relationship between estrogen and benign breast tumors, with a focus on identifying specific pathways and mechanisms underlying tumor development and recurrence. Such investigations are essential to develop effective prevention and treatment strategies. Moreover, it is vital to consider the implications of estrogen-related therapies as they may affect not only tumor growth but also the broader spectrum of breast health.
